# A Step Toward Timely Referral and Early Diagnosis of Cancer: Implementation and Impact on Knowledge of a Primary Care-Based Training Program in Botswana

**DOI:** 10.3389/fonc.2018.00187

**Published:** 2018-05-29

**Authors:** Neo M. Tapela, Michael J. Peluso, Racquel E. Kohler, Irene I. Setlhako, Kerapetse Botebele, Kemiso Gabegwe, Isaac Nkele, Mohan Narasimhamurthy, Mompati Mmalane, Surbhi Grover, Tomer Barak, Lawrence N. Shulman, Shahin Lockman, Scott Dryden-Peterson

**Affiliations:** ^1^Botswana Harvard AIDS Institute Partnership, Gaborone, Botswana; ^2^Division of Global Health Equity, Brigham and Women’s Hospital, Boston, MA, United States; ^3^Department of Medicine, Brigham and Women’s Hospital, Boston, MA, United States; ^4^Department of Social and Behavioral Sciences, Harvard TH Chan School of Public Health, Center for Community-Based Research, Dana-Farber Cancer Institute, Boston, MA, United States; ^5^Princess Marina Hospital, Ministry of Health and Wellness, Gaborone, Botswana; ^6^Department of Pathology, Faculty of Medicine, University of Botswana, Gaborone, Botswana; ^7^University of Pennsylvania, Philadelphia, PA, United States; ^8^Botswana Upenn Partnership, Gaborone, Botswana; ^9^Department of Medicine, Beth Israel Deaconess Medical Center, Boston, MA, United States; ^10^Center for Global Cancer Medicine, Abramson Cancer Center, University of Pennsylvania, Philadelphia, PA, United States; ^11^Harvard TH Chan School of Public Health, Boston, MA, United States; ^12^Division of Infectious Diseases, Brigham and Women’s Hospital, Boston, MA, United States

**Keywords:** cancer early diagnosis, health system delays, primary care, primary care providers, Botswana, sub-Saharan Africa, training

## Abstract

**Introduction:**

Health system delays in diagnosis of cancer contribute to the glaring disparities in cancer mortality between high-income countries and low- and middle-income countries. In Botswana, approximately 70% of cancers are diagnosed at late stage and median time from first health facility visit for cancer-related symptoms to specialty cancer care was 160 days (IQR 59–653). We describe the implementation and early outcomes of training targeting primary care providers, which is a part of a multi-component implementation study in Kweneng-East district aiming to enhance timely diagnosis of cancers.

**Methods:**

Health-care providers from all public facilities within the district were invited to participate in an 8-h intensive short-course program developed by a multidisciplinary team and adapted to the Botswana health system context. Participants’ performance was assessed using a 25-multiple choice question tool, with pre- and post-assessments paired by anonymous identifier. Statistical analysis with Wilcoxon signed-rank test to compare performance at the two time points across eight sub-domains (pathophysiology, epidemiology, social context, symptoms, evaluation, treatment, documentation, follow-up). Linear regression and negative binomial modeling were used to determine change in performance. Participants’ satisfaction with the program was measured on a separate survey using a 5-point Likert scale.

**Results:**

176 participants attended the training over 5 days in April 2016. Pooled linear regression controlling for test version showed an overall performance increase of 16.8% after participation (95% CI 15.2–18.4). Statistically significant improvement was observed for seven out of eight subdomains on test A and all eight subdomains on test B. Overall, 71 (40.3%) trainees achieved a score greater than 70% on the pretest, and 161 (91.5%) did so on the posttest. Participants reported a high degree of satisfaction with the training program’s content and its relevance to their daily work.

**Conclusion:**

We describe a successfully implemented primary health care provider-focused training component of an innovative intervention aiming to reduce health systems delays in cancer diagnosis in sub-Saharan Africa. The training achieved district-wide participation, and improvement in the knowledge of primary health-care providers in this setting.

**Clinical Trial Registration:**

www.ClinicalTrials.gov, identifier NCT02752061.

## Introduction

Cancer is more deadly in resource-rich than resource-limited regions of the world. The mortality-to-incidence ratio of cancers in the United States and the European Union are 0.36 and 0.48, respectively, while they range between 0.66 and 0.70 in World Health Organization’ Western Pacific, Southeast Asia, and Africa regions (1, 2). As an example, overall, 5-year survival for breast cancer ranges from 88% in the US, 64% in South Africa, and 56% in Uganda, and significantly less than this in many parts of Africa (3–6). This glaring disparity in outcomes is due in part to late diagnosis of cancer (2, 4), which corresponds with poorer prognosis.

Patient, provider, and health system factors contribute to late diagnosis of cancer (7). Fears, stigma, limited cancer awareness and fatalistic beliefs (8, 9), male sex (10), spiritual and traditional beliefs (11–14), as well as low socioeconomic position (9, 11, 15) and difficulty navigating the health system are notable patient-related factors affecting delays. Health systems and provider factors include shortage of health-care providers, limited cancer knowledge and skills (12, 14, 16), lack of diagnostic tools and technologies including pathology services (17, 18), diagnostic errors (4), type of cancer (19), geographic distribution of services, and delayed referrals and poor coordination between facilities (8). Although much of the literature describes patient-related delays, evidence suggests that health system delays may play a greater role in late diagnosis (4, 20, 21), and few studies have implemented and evaluated interventions aimed to address health system factors.

In Botswana, a middle-income country of two million people, approximately 70% of cancers are diagnosed at late stage (unpublished, National Non-Communicable Diseases Program data) despite efforts to increase cancer screening and provision of treatment of major cancers to citizens free of charge. We previously found that median time from symptom onset to first presentation at a health facility was 29 days (IQR 0–185), while median time from first health facility visit to specialty cancer care was 160 days (IQR 59–653) (10). Therefore, we sought to implement and evaluate a comprehensive quality improvement project to facilitate earlier cancer detection, particularly targeting health system and provider-related factors.

The Potlako initiative is an implementation study in Kweneng-East district in Botswana that aims to enhance timely diagnosis of cancer through a package of (a) training primary care clinicians to identify, manage, triage, and refer patients with cancer-related symptoms, (b) developing a standardized referral algorithm, (c) introducing a nurse coordinator role to support patients and clinicians across facility levels to navigate the health system, and (d) providing transport assistance for vulnerable patients (22). To the best of our knowledge Potlako, which means “hurry” in Setswana, is the first study in sub-Saharan Africa to evaluate a multi-component intervention package that addresses contributors to both patient and health system delays to improve timely diagnosis of cancer. Here, we describe the implementation and early outcomes of the training component of this intervention. Results from other Potlako components will be assessed and reported after study completion.

## Materials and Methods

### Study Setting

Botswana has a relatively well-resourced health-care system, with primary health-care services available for free to all citizens and over 95% of residents residing within 8 km of a health facility (23). There are 38 doctors, and 272 nurses/midwives per 100,000 population (24). The country has a network of over 600 of primary health-care facilities (health posts and primary clinics) each staffed by at least one general nurse (with at least 2 years diploma post-secondary training). Government is the leading source of health-care financing covering 65% of total health expenditure (25), and total health expenditure per capita is Int’l $ 87 (26). Treatment of cancer, including chemotherapy, systemic therapy, surgery, and radiotherapy is available for free to all citizens who meet clinical indication at tertiary facilities located in the two largest cities in the country.

Patients with cancer are referred for treatment at Princess Marina Hospital, a 500-bed facility that is one of the two national referral hospitals providing oncology specialty services. Cancer pathology-based diagnosis and treatment (chemotherapy and systemic, surgery) are available at public facilities for free to citizens; radiotherapy at a private hospital is also available for free for patients referred through the public facility system.

**Figure 1:**
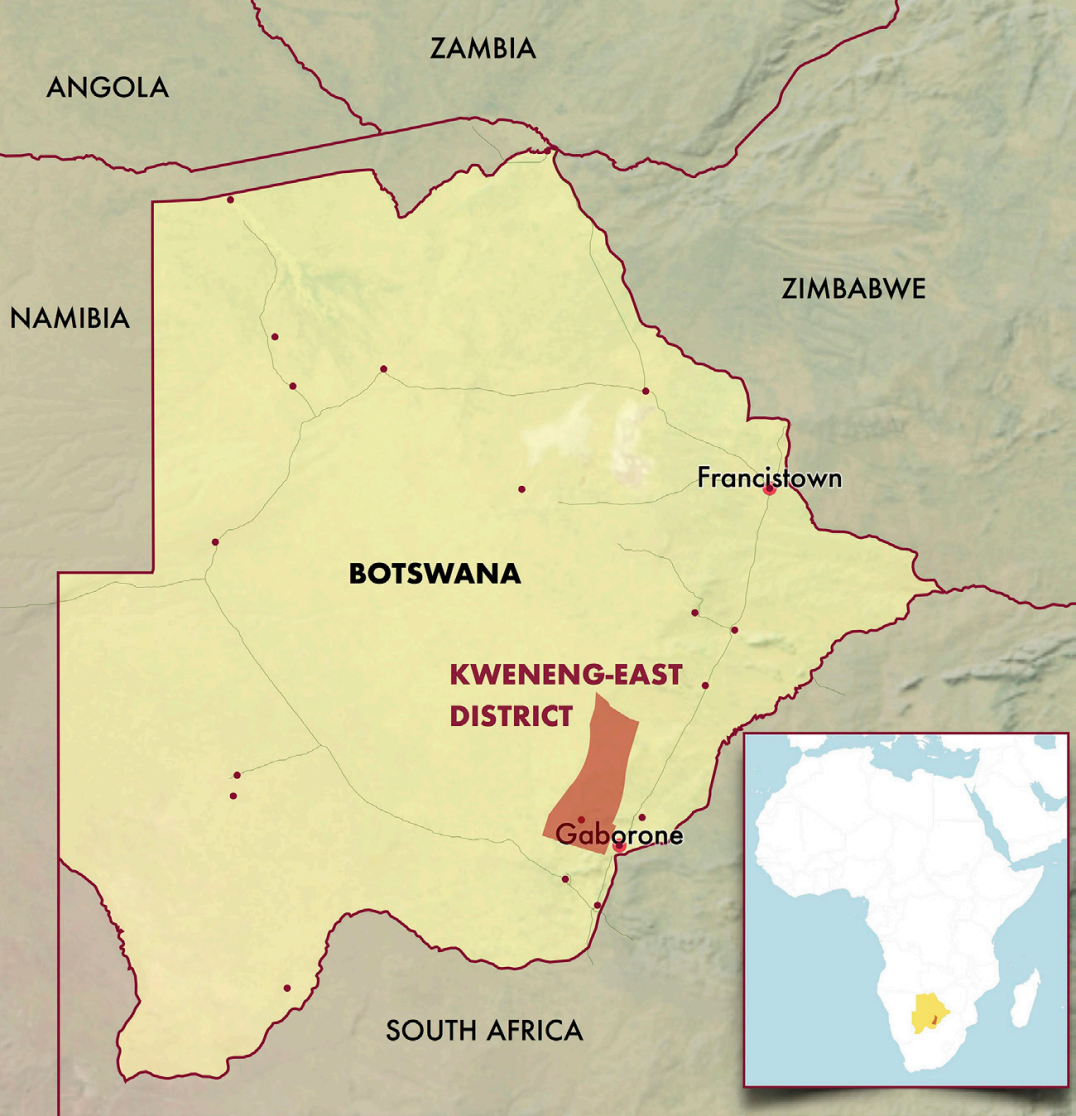
Map of Botswana and Kweneng-East district.

Potlako initiative is based in Kweneng-East District, one of Botswana’s 27 health districts located approximately 50 km west of Botswana’s capital city Gaborone. The health district serves 150,131 residents (27) who live in a range of settings including semi-urban to rural remote cattle posts (Figure 1). Potlako is a district-wide initiative involving participation of all 35 primary care clinics (21 health posts, 14 primary clinics) as well as a primary hospital and a general (district) hospital in the district.

### Trainees and Process of Invitation

The training component of Potlako targeted health-care providers, particularly nurses, practicing at primary care clinics who typically are the first point of contact for adults with undiagnosed cancer symptoms. Providers from all public facilities in Kweneng-East District were invited to participate, including the HIV/infectious diseases care clinic (IDCC), sexual and reproductive health clinic, and general medical and surgical outpatient clinics (OPD). For the two district hospitals, providers from the inpatient medical, surgical, and gynecologic wards were also invited. Overall 170 health-care providers were invited to the training, with a goal of training at least 40% of those working in primary clinics and at least 25% of those at relevant outpatient clinics and wards in the hospital.

A dedicated process of stakeholder engagement and participation in planning was followed. A series of meetings were held, first involving the head of district management team and facility managers to identify facility-based focal points at each facility. Then focal points worked with managers to identify individuals suitable for training given specified criteria such as working in the relevant units and size of facilities. Training dates were also set according to preferences expressed by facility and district leadership. Letters of invitation were emailed and faxed, and subsequently followed up by phone-call confirmation of names of those who would be attending.

### Curriculum Development and Trainers

The overall objective was to empower primary health-care providers with increased knowledge on how to identify, provide primary care, and promptly and appropriately refer patients who may have cancer (Boxes 1 and 2). We developed an intensive 8-h short-course program, employing multi-method format including participatory didactic slide-based presentations, role-playing demonstrations, case studies, small group discussions, self- and group-reflection on clinical experiences, and open question and answer sessions.

Box 1 **Overview of Potlako health-care provider learning objectives.**

**Table:** 

**Overall objective**
The training placed an emphasis on concrete and practical steps health-care providers can take to (a) Interview patients and evaluate them on physical exam, (b) Care for patients with suggestive signs and symptoms of cancer at primary care level, including through counseling, pain management, and other supportive measures, triage and stabilization, and (c) Refer appropriately with proper documentation, communication, and transportation arrangements as needed.

**Learning outcomes**
By the end of the training participants should be able to: Describe burden of major cancers in the world and in Botswana, key risk factors, and the types of cancer services available in Botswana. Identify patients who may have cancer, based on assessing for suggestive symptoms in detailed history taking and assessing for suggestive signs on physical exam. Differentiate between triage statuses of patients suspected to have cancer, based on considerations such as performance status, already incurred health system delays, presence of general medical or suspected oncologic emergency. Use triage status to determine where, when and how to refer patients appropriately. Provide appropriate initial medical management at primary care clinic, including stabilization of emergent patient and evaluation using services available at primary care level Discuss with patients the possibility of cancer as a cause of symptoms and the general process of evaluation and follow up Explain and provide education and support relevant to common cancer myths and stigma issues, with sensitivity toward individual, religious, and cultural beliefs List relevant services and clinics available at Scottish Livingstone Hospital (district) and Princess Marina Hospital (tertiary) available for diagnostic evaluation of cancer (NB. key contact information and clinic scheduling were also provided at the training)

Box 2 **Training session topics and format.**

**Table:** 

Session title	Format	Session details
Cancer in Botswana	Didactic slide presentation	Overview of the epidemiology of cancer in Botswana, including most common types, prevalent risk factors, and trends in incidence cases over time. Services for screening, diagnosis, and treatment of cancer available in Botswana’s public sector were reviewed including facility names and key contacts

Cancer myths and stigma	Interactive session including role plays	Discussion of common myths and stigma related to cancer and approaches on how to provide education and counseling in culturally sensitive and supportive manner

Basic principles of cancer	Didactic slide presentation	Basic principles of cancer pathophysiology, available strategies for primary and secondary prevention, the role of pathology-based diagnosis and cancer staging, and major categories of cancer treatment

Signs and symptoms of cancer	Didactic slide presentation with clinical images and clinical vignettes	Symptoms and signs that can be associated with cancer, how to pick up constellations of them, and differential diagnosis to consider

Overview of major cancers	Didactic slide presentation with clinical images	For the most common cancers, review of clinical presentation, diagnosis and staging necessary, treatment options, and unique considerations such as post-mastectomy arm edema in breast cancer

Cancer-related emergencies	Didactic slide presentation with clinical images and clinical vignettes	Key signs and symptoms that may be seen in medical emergencies that can occur in cancer patients such as neutropenic fever and airway obstruction. How to recognize these as well as initial acute management and emergent transfer

Palliative care for cancer patients	Didactic slide presentation, with clinical vignettes	Defining what palliative care is, what it is beyond pain management, and its role across the spectrum of illness and beyond death. The role of primary-care providers in delivering palliative care

Encountering patients	Didactic slide presentation with clinical vignettes	Presentation of criteria to consider in triaging patient as stable, urgent, or emergent. For stable patients determining where to refer them and time interval of follow-up, by navigating referral algorithm

Case studies practice	Group work clinical vignettes, practice, and role plays	Opportunity to synthesize concepts across all previous sessions, where participants practice approaches of how to assess, manage, and refer patients in front of them, and what to say to patients. Clinical vignettes include patient presenting to clinic with sepsis, another with symptoms and signs common to TB and lymphoma, and patient

The curriculum was developed by a multidisciplinary team with experience working in Botswana (two oncologists, one oncology fellow, one medical officer, one infectious disease specialist, one internal medicine resident, one family medicine resident, one internal medicine specialist, one pathologist, one palliative care specialist, one public health officer, and two nurses). Consultation and inputs from the team of experts were obtained through workshops, individual discussions, and email communication, to reach consensus regarding (a) competencies of health-care providers relevant to cancer early detection within study context and relevant learning outcomes, (b) general teaching and learning strategies, (c) training material being developed, and (d) training evaluation tool (described below). Curriculum content was adapted to the Botswana context using training material that had been used for similar purposes in Rwanda’s national baseline cancer training program jointly developed by Rwanda Ministry of Health, Partners in Health and Dana Farber/Brigham and Women’s Cancer Center (28, 29). Adaptation employed an iterative review process incorporating inputs from the literature and training team expertise and lessons learned in structuring Botswana’s HIV training program (KITSO) for primary-care providers (30).

The curriculum was delivered as a 1-day program and was administered five times in April 2016. Most of the trainers facilitating the lectures and activities (Table 2) also contributed to curriculum development. All participants received printed materials of lecture slides, referral algorithms, and discussion topics.

### Training Evaluation

A single answer multiple-choice format pre- and posttest was developed to evaluate knowledge gained and achievement of learning objectives of the training program (Table 1). While we did not employ a formal consensus building technique, development of the training pre/posttest was informed by literature review, previous experience with similar trainings in rural Rwanda, and involved an iterative process of consultation and revisions by a multi-disciplinary panel of experts (the same individuals who had been involved in the broader curriculum development). Inputs from experts were obtained through workshops, individual discussions, and over email. Items selected for inclusion were those that assessed immediate impact of training in accordance with the first two levels of Kirkpatrick’s training evaluation framework (31): (1) reaction and (2) acquisition of knowledge, attitudes, and skills. Selection of questions emphasized diversity across two broad domains of knowledge and management, and corresponding eight sub-domains: pathophysiology, epidemiology, social context, symptoms, evaluation, treatment, documentation, follow-up. Prior to finalization of the pre/posttest, inputs were obtained from the team regarding clarity and specificity of questions, and agreement on the correct answer.

**Table 1:** Sample questions from pre- and posttests.

Domain or sub-domain	Sample question
**Knowledge**	

Epidemiology	Which of the following describes the overall trend of cancer in Botswana? (a) The number of new cases of Kaposi sarcoma is increasing, while the number of new cancers of all other cancers is generally decreasing (**b**) **The number of new cases of Kaposi sarcoma is decreasing, while the number of new cancers of all other cancers is generally increasing** (c) The number of new cases of Kaposi sarcoma is staying the same, while the number of new cancers of all other cancers is generally increasing (d) We don’t know anything about the number of new cases of cancer in Botswana

Pathophysiology	Which of the following best describes how cancer develops? (**a**) **A damaged cell divides in an uncontrolled manner** (b) A normal cell grows into a mass (c) A normal cell travels through the blood stream to other parts of the body (d) A cell spreads from one person to another person through touching

Sociocultural context	You are seeing a patient in clinic who has been diagnosed with colon cancer. He tells you that he has not followed up at Marina over the past few months because he has been seeing a traditional healer and believes that he will be cured this way. While there is no single right answer, the following is one helpful approach in counseling this patient: (a) Mention to the patient that you do not know anything about cancer and its treatment, as you are not a cancer specialist (b) Respectfully avoid discussing spiritual and traditional beliefs, as these are very personal and can never be aligned with western medical treatment (**c**) **Point out that while spiritual wellbeing and traditional beliefs are important, they should not be a substitute for proven medical treatments for cancer** (d) State that traditional medicines are perfectly safe and do not interfere with medical therapies such as chemotherapy

Symptoms	Which of the following is a symptom that might be seen in cancer? (a) Weight gain despite regular exercise (b) Increased energy (c) Increased appetite (**d**) **Night sweats**

**Management**	

Evaluation	*You are at Phuthadikobo clinic and you see a 49-year-old woman who is complaining of headache. Upon history taking, you find that the headache has been on and off for a few weeks. She has four children and experienced menopause about 5 years ago. Most recently, she has noticed some bloody discharge from her vagina and wonders if her period might be coming back. She says that her appetite might be up and down. She denies: fever, confusion, weight loss, or weakness*. What is important to include in your evaluation of this patient at clinic today? (a) Pap smear (**b**) **Pelvic speculum exam** (c) Lumbar puncture (d) No further evaluation at clinic is necessary, refer patient immediately to SLH

Counseling/documentation	You have just seen a 45-year-old male patient at Lekgwapheng clinic, who is showing some signs suspicious for cancer. He walked to clinic today accompanied by his wife. You would like to refer him to Scottish Livingstone Hospital (SLH) for further evaluation. What documentation should you fill out at the end of the visit today? (**a**) **OPD card, clinic cancer register, and referral form** (b) OPD card, referral form, and biopsy requisition form (c) Clinic cancer register and referral form (d) OPD card, clinic cancer register, and biopsy requisition form

Treatment	A 33-year-old HIV-positive male who has recently been diagnosed with lymphoma comes to see you at Lephepe clinic. His HIV has been well controlled for several years. He has been receiving chemotherapy at Marina, with the most recent treatment cycle 1 week ago. He is complaining of fever, cough, and fatigue for 3 days. His vital signs are: temperature 39.3°C, blood pressure 90/60, pulse 128 beats per minute, respirations 25/min, saturating 100% on room air. What are the next most important steps in managing this patient? (a) Give oral antibiotics, order chest X-ray, and ensure follow up to clinic in 2 weeks (b) Give IV antibiotics, and refer same day to the nearest IDCC (c) Give IV antibiotics and ORS, schedule follow-up appoint to clinic in 1 week and counsel the patient on symptoms to come back immediately to clinic for (**d**) **Give IV antibiotics, and refer same day to SLH A&E**

Follow-up	You have just seen a 45-year-old male patient at Lekgwapheng clinic, who is showing some signs suspicious for cancer. He walked to clinic today accompanied by his wife. You would like to refer him to Scottish Livingstone Hospital (SLH) for further evaluation. In what way might you arrange follow up to identify in a timely and feasible manner any challenges with your referral of this patient and others like him? (a) Ask the patient to call you after the SLH visit (b) Call the patient every week to find out if he was seen at SLH (**c**) **Schedule a follow-up appointment at Lekgwapheng clinic, for 1 month from today** (d) Call the SLH OPD every month to find out if the patient was seen

*Correct answers in bold*.

In response to feedback from participants during the first 2 days of training, trainers (who included members of the curriculum development team) identified the need to refine some of the questions to put less emphasis on the epidemiology, and more emphasis on practical information on evaluation, treatment and counseling as well as follow-up. The format of some of the questions was also revised from stand-alone questions to clinical vignettes containing a series of questions. This resulted in change in content of 14 questions. Given this change, analysis for pre/posttest scoring were performed and presented separately as test version A (first and second trainings), and version B (third, fourth, and fifth trainings).

Pre/posttests were administered on paper, with trainees asked to generate an anonymous ID code to allow for matching of answers across pre/posttest time points. The posttest was identical to the pretest administered on any given training day. Following completion of posttest, questions were reviewed with open discussion of correct answers and questions that were particularly challenging. Trainees were also asked to complete an anonymous satisfaction form in order to provide feedback on the quality of execution of the training including relevance to day-to-day work, clarity of content, and general training logistics. On a daily basis, research assistants transcribed the pre- and posttests from paper into excel spreadsheet that had pre-programmed data quality checks and scoring. 10% of tests were audited by NT and MP.

### Statistical Analysis

Correct responses were summed and overall scores were calculated as the total percentage of correct responses. Domain and sub-domain scores were also calculated. A score of 70% or greater was considered passing. Descriptive statistics were used to characterize counts of correct responses, overall score percent, and change. We used the Wilcoxon signed-rank test to compare differences between test versions and scores. Because of the changes across test versions and significant differences in scores between versions, data for each version of the exam are presented separately. We used a panel design and ran both linear regression (on overall percent score) and negative binomial (on correct count) models clustered on the individual and controlling for the test version to determine the change in performance. Analyses were performed using Stata (Version 14, College Station, TX, USA).

### Ethical Review

Project was reviewed and approved by the institutional review boards of the Botswana Ministry of Health and Wellness and the Harvard T.H. Chan School of Public Health. Additional approvals were obtained from the participating health facilities. Requirement for written informed consent was waived given the minimal risk of the study, and to reduce risk of reputational harm based on test performance.

## Results

### Participation

At the beginning of the study period, Kweneng-East had 134 nurses and 7 rotating general practitioner doctors staffing the primary clinics; the two hospitals had 26 doctors and 327 nurses on their rosters. We invited 170 providers to the program, with a goal of training at least 40% of those working in primary clinics to ensure availability of trained personnel even with routine staffing turn-over. A total of 176 trainees attended with 100% of all Kweneng-East public facilities represented and 60% of general nurses, midwives, and family nurse practitioners working at the primary clinics attending the training. 111 (63%) of trainees were nurses, 36 (21%) were midwives, 7 (4%) were family nurse practitioners, and 19 (11%) were general practitioner doctors. 87 of the trainees (49.4%) were from primary clinics, while 22 (12%) were from outpatient clinics at the primary and district hospitals, 52 (30%) from the inpatient wards at those hospitals and 12 (7%) were from unspecified units at the hospitals.

### Overall Knowledge Gains

All attendees completed pre/posttests. There were significant differences between the scores on different test versions (Table 2). Among the 79 trainees who completed test version A, the mean change was 12.9 percentage points. Among the 97 who took test version B, the mean change was 20.0 percentage points. The pooled linear regression results for the overall score showed an increase of 16.8 percentage points on the posttest controlling for the test version [95% confidence interval (CI): 15.2–18.4, *p* < 0.001].

**Table 2:** Pre- and posttest scores, by domain and subdomain.

	Pretest Median (IQR)	Posttest Median (IQR)
**Test version A (***N*** = 79)**		
Total score*	68.0 (12.0)	84.0 (12.0)
Knowledge***	69.2 (15.4)	84.6 (15.4)
Epidemiology*	100.0 (0.0)	100.0 (0.0)
Pathophysiology*	75.0 (50.0)	75.0 (25.0)
Social context***	75.0 (25.0)	75.0 (25.0)
Symptoms***	66.7 (33.3)	100.0 (33.3)
Management***	66.7 (16.7)	83.3 (8.3)
Evaluation***	66.7 (0.0)	100.0 (0.0)
Documentation***	66.7 (33.3)	100.0 (0.0)
Treatment	66.7 (33.3)	66.7 (33.3)
Follow-up**	33.3 (33.3)	66.7 (33.3)
**Test version B (***N*** = 97)**		
Total score	64.0 (20.0)	88.0 (12.0)
Knowledge***	66.7 (16.7)	91.7 (16.7)
Epidemiology***	100.0 (50.0)	100.0 (0.0)
Pathophysiology***	66.7 (66.7)	66.7 (33.3)
Social context***	66.7 (66.7)	100.0 (33.3)
Symptoms***	75.0 (50.0)	100.0 (0.0)
Management***	61.5 (15.4)	84.6 (15.4)
Evaluation***	0.0 (50.0)	100.0 (50.0)
Documentation***	100.0 (25.0)	100.0 (25.0)
Treatment***	50.0 (50.0)	100.0 (50.0)
Follow-up***	60.0 (40.0)	80.0 (40.0)

*p ≤ 0.05; **p ≤ 0.01; ***p ≤ 0.001.

We used an arbitrary cut-off score of 70% as the threshold for passing. Prior to the training, less than half of the trainees passed the assessment test (49% version A, 33% version B). Following the training, nearly all participants passed (89% version A, 94% version B). Overall, about half of the sample improved from below the threshold on the pre-test to above the passing score (92, 52%) after the training. However, 13 (7%) did not improve their scores above passing. All but two participants with passing scores on the pre-test also passed the posttest.

### Knowledge Gains Across Sub-Domains

We analyzed improvement within two domains and saw significant improvements in both the knowledge and management domains on both test versions (Table 2). Participants completing test version A demonstrated improvement in the following subdomains: epidemiology (*p* = 0.04), pathophysiology (*p* = 0.019), social context (*p* < 0.0001), symptoms (*p* < 0.0001), evaluation (*p* < 0.0001), documentation (*p* < 0.0001), and follow-up (*p* = 0.0029). There was no difference in the treatment subdomain. Participants completing test version B demonstrated improvement in all subdomains: epidemiology (*p* < 0.0001), pathophysiology (*p* < 0.0001), social context (*p* < 0.0001), symptoms (*p* < 0.0001), evaluation (*p* < 0.0001), documentation (*p* < 0.0001), treatment (*p* < 0.0001), and follow-up (*p* = 0.0003).

### Trainee Satisfaction With Training Program

Ninety-six percent (169) participants completed the satisfaction survey. Overall, participants reported a high degree of satisfaction with the training program. On a 5-point scale, participants rated the format in which the training was delivered, including the balance between lectures and interactive sessions with a mean score of 4.5 (range 1–5). They rated the content of the training, including key learning points and topics covered, with a mean score of 4.5 (range 3–5). Participants rated the usefulness of the training to their job, including how they would be able to utilize the lessons from it in their day-to-day work, with a mean score of 4.7 (range 3–5). Of 169 respondents, 166 (98.2%) stated that they would be interested in attending a follow-up training.

### Training Material and Tools

A set of training material including didactic power-point slides, case studies/scenarios, trainer’s notes, and pre/posttest has been developed. These have been placed in repository that is accessible for future trainings and adaptation for other related trainings.

## Discussion

We successfully implemented the health-care provider training component of an innovative intervention aiming to reduce health systems delays in cancer diagnosis, had high district-wide attendance, and demonstrated gains in knowledge among participants. Knowledge gaps identified and addressed included in the areas (sub-domains) of understanding better a patient’s social context, cancer-associated symptoms, and primary-care based patient evaluation, and follow-up.

### Curriculum Development

Development of the curriculum of this training leveraged South–South collaboration and use of material and experience developed in the region including in Rwanda (24, 25) and national structured HIV training program targeting primary care health-care providers that facilitated Botswana’s rapid implementation of the continent’s first national anti-retroviral treatment program (30). Training material is available for future implementation, adaptation, and sharing and there is a need to strengthen avenues for best practices sharing of these and training approaches, such as have been initiated by American Society of Clinical Oncology and the Kenya-based Academic Model Providing Access to Health care consortium (32).

There have been many more studies on training to improve early detection of disease focusing non-communicable diseases (NCDs) other than cancer, and those involving cancer have been specific to a type of cancer such as oral cancer in Asia and cervical cancer in Africa (33–36). The training program presented here is among the few that we are aware of that covers cancers broadly and concepts in signs, symptoms, triage, referral in consideration of a group of major cancers. It is also one that focuses on history and physical exam, available at the primary care level in any setting, rather than reliant on need for additional technology or equipment. While basic, this training is indeed fitting for primary care and low technology setting and employing an integrated approach. The training also serves as a pilot and first building block for a core module on cancer early detection, to which other components such as clinical breast exam practicum can be added in the future.

### Training Participation and Stakeholder Engagement

The positive response to training invitations reflects an interest in learning opportunities about cancers and a knowledge gap identified by facility managers. The high rate of participation was likely the result of active stakeholder engagement in the planning process.

### Impact of Training on Knowledge

We found a statistically significant improvement in overall and across several sub-domains including in cancer-associated symptoms, appropriate evaluation, referral, and follow-up. These are notable as medical training institutions in Botswana, as with those in many LMICs (32), have traditionally not included significant cancer content in their undergraduate curriculums. Furthermore, Botswana’s experience with high prevalence of HIV, which peaked at 27% in 2001 (37), and the successful scale of up a structured training (30) has resulted in most health-care providers being competent in diagnosis and management of major infectious diseases while seldom considering cancer and NCDs in their differential diagnosis, much less managing appropriately. This knowledge gap is increasingly important to address given the changing landscape of longer life expectancy, unhealthy lifestyle habits, and indeed the excess risk of cancer introduced by HIV. The described training focuses on tackling this gap at patient’s first encounter with the health system, the primary care level.

### Limitations

This analysis is not without its limitations. First, given the revision of pre/posttest administered on the last 3 days of the training, we were not able to meaningfully analyze data across all 5 days of the training period. We thus present test scores during the first 2 days of training and the last 3 days of training separately. We were unable to collect information on demographics and other characteristics of trainees and, therefore, could not investigate differences across sub-groups of trainees. We found a statistically significant 16.8% absolute difference between pre- and posttesting scores, this was not as large an improvement as observed in other studies (38, 39). This may indicate that the test questions could have been designed to be more challenging; to the best of our knowledge, there are no relevant validated testing tools that might have been applied. Furthermore, while pre/post testing and setting of an arbitrary passing threshold is informative in understanding knowledge gained during the training, the ultimate assessment is whether the learning is retained and translates into improved care for patients who could have cancer.

While our training intervention was effective with respect to immediate learning outcomes, we recognize that this is not sufficient to translate to improved practices as has been described in many studies (40–42). Other than knowledge and skills, there are other determinants of health-care provider performance as it related to early detection, such as provider’s motivation, attitudes and perceptions regarding responsibilities, and work environment (40). These would need to be evaluated and addressed in further studies. Additionally, there are patient and health system factors to take into account, such as patient awareness and socioeconomic barriers, and health system funding, leadership, policies, and guidelines in place overall absorptive capacity personnel and infrastructure (43, 44). Thus, we present here only a piece of the puzzle, so to speak, to achieve earlier detection of cancer in our context. The broader Potlako initiative seeks to address these other factors as well, including through case management mentorship supports, job aids, coordination of visit scheduling and pathology results, navigation, and transport support for patients. While a comprehensive cancer early detection strategy and program does not currently exist in Botswana, there are various policies and guideline developments that support cancer early detection. Early detection is listed as a component of Botswana’s Essential Health Services Package, a national cervical cancer screening program has been in existence since 2012 (34) and roll out is ongoing, the country’s first national Primary Health-care guidelines, which include protocol for early detection of breast cancer were endorsed in 2016, and development of national treatment guidelines for specific cancers is underway.

## Conclusion

This description of the development, implementation, and preliminary knowledge gains from a health-care provider training demonstrate an avenue by which primary care health-care providers can be engaged in efforts to diagnose cancer earlier. This report is particularly timely given increasing global discourse on leveraging the primary care platform in strengthening health systems, and in more efficiently using limited health resources in low- and middle-income countries. Further studies are needed to assess and address the other key determinants of health-care provider performance related to early diagnosis, such as provider’s motivation, attitudes and perceptions regarding responsibilities, and work environment. Our description of approach and findings thus far are a first step in this inquiry, which may inform Botswana’s cancer early detection approaches facilitated by implementation of Primary Health-care guidelines. Findings may also be relevant to other settings seeking to improve cancer early detection and quality of service delivery through training of primary care providers.

## Availability of Data and Material

The datasets used and/or analyzed during the current study are available from the corresponding author on reasonable request.

## Ethics Statement

Project was reviewed and approved by the institutional review boards of the Botswana Ministry of Health and Wellness and the Harvard T.H. Chan School of Public Health. Additional approvals were obtained from the participating health facilities. Requirement for written informed consent was waived given the minimal risk of the study, and to reduce risk of reputational harm based on test performance.

## Author Contributions

NT conducted literature review, contributed to study conception and design, supported cleaning, analysis and interpretation, and led writing and critical review of the manuscript. MP conducted literature review, performed data analysis, and contributed to writing and critical review of manuscript. REK supported data analysis and contributed to critical review and editing of manuscript. SD-P and SL contributed to the conception, analysis interpretation, and critical review of the manuscript. KB, KG, and IN contributed to data collection and data cleaning, and critical review of manuscript. IIS, MN, MM, SG, TB, and LS contributed to interpretation of findings, organization and critical review of manuscript.

## Conflict of Interest Statement

The authors declare that the research was conducted in the absence of any commercial or financial relationships that could be construed as a potential conflict of interest.
